# Slight religiosity associated with a lower incidence of any fracture among healthy people in a multireligious country

**DOI:** 10.1186/s13030-023-00265-6

**Published:** 2023-02-09

**Authors:** Daiki Kobayashi, Hironori Kuga, Takuro Shimbo

**Affiliations:** 1 Division of General Internal Medicine, Department of Medicine, Tokyo Medical University Ibaraki Medical Center, Ibaraki, Japan; 2 Fujita Health University, Toyoake, Japan; 3 Department of General Medicine, Faculty of Medicine, Juntendo University, Tokyo, Japan; 4 National Center for Cognitive Behavior Therapy and Research, National Center of Neurology and Psychiatry, Kodaira, Japan; 5 Department of Medicine, Ohta Nishinouchi Hospital, Koriyama, Japan

**Keywords:** Fracture, Japan, Large scale, Longitudinal study, Religion, T score

## Abstract

**Background:**

The aim of this study was to evaluate the association between the degree of religiosity and subsequent fractures and a decrease in bone mineral density in a Japanese population.

**Methods:**

We conducted a retrospective longitudinal study at St. Luke’s International Hospital in Tokyo, Japan, from 2005 to 2018. All participants who underwent voluntary health check-ups were included. Our outcomes were any fractures and the change in T-score from baseline to each visit. We compared these outcomes by the self-reported degree of religiosity (not at all; slightly; somewhat; very) and adjusted for potential confounders.

**Results:**

A total of 65,898 participants were included in our study. Their mean age was 46.2(SD:12.2) years, and 33,014(50.1%) were male. During a median follow-up of 2,500 days (interquartile range (IQR):987–3,970), 2,753(4.2%) experienced fractures, and their mean delta T-score was -0.03%(SD:18.3). In multivariable longitudinal analyses, the slightly religious group had a statistically lower adjusted odds ratio (AOR) for a fracture than the nonreligious group(AOR:0.81,95% confidence interval(CI):0.71 to 0.92).

**Conclusions:**

We demonstrated that slightly religious people, but not somewhat or very religious people, had a lower incidence of fracture than nonreligious individuals, although the T-scores were similar regardless of the degree of religiosity.

**Supplementary Information:**

The online version contains supplementary material available at 10.1186/s13030-023-00265-6.

## Key messages

- ・Slightly religious people had a lower incidence of any fractures.
- ・Somewhat or very religious people didn’t have a lower incidence of any fractures
- ・The T-scores were similar regardless of the degree of religiosity.

## Introduction

Regardless of the type of religion, religious people have been shown to have better mental and physical health outcomes [1]. As a benefit of religiosity, religious people have also been shown to have longer life expectancies than less religious people [2–5]. However, in terms of each physical disease and its risk factors, the evidence is still mixed. For instance, systematic reviews have indicated that the incidence of cardiovascular disease and its mortality were lower among religious populations [5], but large cohort studies and reviews found the opposite [6, 7]. Among the cardiovascular risk factors, our previous study found a lower risk of future diabetes but did not reveal changes in hypertension or dyslipidemia among religious people [8]. Unfavorable evidence for religiosity is dominant in some unhealthier conditions, such as obesity/overweight [7, 9–11]. Similarly, in terms of mental health, such as depression, the evidence is mixed [1]. Further evidence is required to evaluate these associations.

Although osteoporosis/fracture is considered to be one of the important health conditions of elderly individuals, a very limited number of studies about the association between religion and osteoporosis/fracture have been published. One cross-sectional study showed different prevalences of osteoporosis by the type of religion among women [12]. However, detailed studies on this topic are scarce. For osteoporosis/fractures, a fracture would be considered a better outcome than osteoporosis. The degree of religiosity or religion among different populations should be evaluated.

The aim of this study was to evaluate the association between the degree of religiosity and subsequent fractures and decreases in bone mineral density in a Japanese population.

## Methods

We conducted a retrospective longitudinal study at St. Luke’s International Hospital, a large teaching hospital in Tokyo, Japan, from 2005 to 2018. We included all participants who underwent voluntary health check-ups at the center of preventive medicine in the hospital from 2005 to 2012. The purpose of health check-ups is to screen for and manage chronic diseases, including malignancy, hypertension, diabetes and osteoporosis. We excluded participants who had a prior history of any fractures at baseline visits based on their medical records at the hospital and self-reports. Our primary outcome was any fracture during follow-ups up to December 31, 2018, and the secondary outcome was the change in T-score from baseline to each visit. We compared these outcomes by the self-reported degree of religiosity, adjusting for potential confounders.

St. Luke’s Ethics Committee Institutional Review Board approved this study (approval number: 18-R203. Comprehensive approvals for studies about social habits).

### Fractures and Delta T-scores

Our primary outcome was any fracture during the follow-up period. Information about a fracture was obtained based on electronic medical records at the hospital and self-reports at the time of each follow-up visit. The fracture information at the hospital contained the site of the fracture and its international classification of disease 10 (ICD-10) codes [13], whereas that from a self-report had only the history of a fracture. Those who had fractures multiple times during the study period only had their first episode of a fracture recorded as an outcome.

The T-scores were calculated based on the bone mineral density (BMD) in comparison to young adult mean (YAM) values based on the World Health Organization (WHO) criteria [14]. Participants had the BMD of their radial bone measured as a part of their health check-ups at each follow-up visit by dual-energy X-ray absorptiometry (DEXA) DCS-6EX-3 by ALOKA (Tokyo Japan). The T-scores are expressed not as the standard deviation (SD) but as the percentage (%).T-scores of 100% and 70% in Japan are equivalent to 0 SD and -2.5 SD, respectively, according to the WHO criteria [15]. The delta T-score was defined as the change in the T-score from baseline at each visit.

### Degree of religiosity

From 2005 to 2012, all participants were asked about their religiosity by responding to the following questionnaire at each follow-up visit: “Are you religious? (translated from Japanese)”. Participants chose one of the following responses: not at all, slightly, somewhat, and yes. We categorized them into four groups: not religious at all, slightly religious, somewhat religious and very religious according to their response to this question. These responses were obtained at every follow-up visit and involved a time-dependent variable. We considered “none” as the reference group for the analyses.

### Confounders

We obtained the following information to consider potential confounders in the analyses: participant demographics, health habits, comorbidities, and treatment for osteoporosis. The demographics included age, sex and body mass index (BMI). BMI was calculated by trained staff who measured the participant’s height and weight at each visit. The BMI was categorized into three groups based on WHO criteria for Asians [16]: underweight (BMI < 18.5 kg/m^2^), normal (18.5–24.9 kg/m^2^) or obese/overweight (≥ 25.0 kg/m^2^). Participants were asked about their health habits at each visit as well: alcohol consumption (abstainer, occasional drinker or regular drinker), smoking status (never, former or current) and exercise habit (almost none, 1–2 times a week, 3–5 times a week, or almost all days). Information about comorbidities, including a current diagnosis of hypertension, diabetes and dyslipidemia, was based on self-reports. The history of depression and its treatment status were also taken. In addition, information about pharmacological treatment status and each medication taken for osteoporosis was obtained based on medical records or self-reports. These potential confounders were collected at every follow-up visit and considered to be time-dependent covariates.

### Statistical methods

We obtained descriptive statistics on baseline characteristics and outcomes by baseline degree of religiosity. Next, multivariable longitudinal analyses were performed, adjusting for potential confounders. The generalized estimating equation (GEE) with a binomial distribution and logit link function was used for the incidence of any fracture, and a mixed effects model was used for the delta T-score. We applied different confounders for adjustment for different multivariable models: model 1 was adjusted for time variables, age and sex, and baseline T-score; model 2 was adjusted for health habits (alcohol consumption, cigarette smoking and exercise) and body mass index in addition to the covariates in model 1; model 3 was adjusted for comorbidities (current diagnosis of hypertension, diabetes and dyslipidemia) in addition to the covariates in model 2; model 4 was adjusted for treatment status for osteoporosis. As subanalyses, a similar approach was applied by stratifying by age (< 50 years, ≥ 50 years) and sex. In addition, we performed similar analyses focusing on hip fracture and vertebral fracture as outcomes.

All analyses were performed by using STATA MP 14.2 in 2019 (STATA Corp., College Station, TX, USA).

## Results

A total of 65,898 participants were included in our study. Their mean age was 46.2 (SD: 12.2) years, 33,014 (50.1%) were male, and their mean baseline T-score was 96.9 (SD: 13.4) %. Table 1 shows the baseline characteristics by the degree of religiosity. In terms of demographic data, religious people tended to be older, female, and obese/overweight. They had positive health habits, such as less regular drinking, less current cigarette smoking, and higher levels of exercise. More current comorbidities were diagnosed among religious people. The prevalence of depression and its treatment status were similar across all religious groups. Regarding fracture-related factors, religious people were more likely to be diagnosed with osteoporosis and be treated for it, but all groups had similar T-scores.

**Table 1:** Baseline characteristics and outcome by baseline degree of religiosity

	Religiosity							
	Not religious at all		Slightly religious		Somewhat religious		Very religious	
	*n* = 16,050		*n* = 25,378		*n* = 17,992		*n* = 6,478	
Outcomes								
Fracture, n (%)	518	(3.2)	933	(3.7)	867	(4.8)	435	(6.7)
Delta T score, % (SD)	0.1	(17.0)	-0.1	(16.9)	-0.1	(16.8)	-0.2	(16.9)
Follow-up periods, days (IQR)	2,346	(843- 3,816)	2,464	(945—3,963)	2,572.5	(1,077—4,032)	2,553	(1,076—4,012)
Demographics								
Age, years (SD)	41.7	(11.2)	45.3	(11.5)	49.3	(12.1)	52.1	(13.0)
Male, n (%)	8,810	(54.9)	13,321	(52.5)	8,080	(44.9)	2,803	(43.3)
Body mass index								
Underweight	1,657	(10.3)	2,385	(9.4)	1,691	(9.4)	576	(8.9)
Normal	11,423	(71.2)	18,164	(71.6)	12,670	(70.4)	4,522	(69.8)
Obesity/overweight	2,968	(18.5)	4,829	(19.0)	3,631	(20.2)	1,380	(21.3)
Health habits, n (%)								
Alcohol consumption								
Abstainer	6,064	(37.8)	9,265	(36.5)	7,263	(40.4)	3,085	(47.6)
Occasional	2,704	(16.9)	4,662	(18.4)	3,276	(18.2)	1,098	(17.0)
Regular	7,282	(45.4)	11,451	(45.1)	7,453	(41.4)	2,295	(35.4)
Cigarette smoking								
Never smoker	9,151	(57.0)	15,074	(59.4)	11,317	(62.9)	4,248	(65.6)
Former smoker	3,174	(19.8)	5,774	(22.8)	4,220	(23.5)	1,444	(22.3)
Current smoker	3,725	(23.2)	4,530	(17.9)	2,455	(13.6)	786	(12.1)
Exercise								
Almost none	7,176	(44.7)	9,649	(38.0)	5,953	(33.1)	2,057	(31.8)
Once to twice a week	5,621	(35.0)	9,846	(38.8)	6,804	(37.8)	2,241	(34.6)
3–5 times a week	1,966	(12.3)	3,704	(14.6)	3,236	(18.0)	1,196	(18.5)
Almost everyday	1,287	(8.0)	2,179	(8.6)	1,999	(11.1)	984	(15.2)
Comorbidities, n (%)								
Hypertension	835	(5.2)	1,774	(7.0)	1,640	(9.1)	745	(11.5)
Diabetes	280	(1.7)	571	(2.3)	510	(2.8)	270	(4.2)
Dyslipidemia	434	(2.7)	992	(3.9)	1,008	(5.6)	450	(7.0)
Depression	447	(2.9)	674	(2.7)	540	(2.9)	212	(3.0)
Medications for depression	167	(1.1)	270	(1.1)	207	(1.1)	86	(1.2)
Fracture related factors								
Baseline T score, % (SD)	96.7	(13.6)	96.8	(13.4)	97.0	(13.2)	96.9	(13.2)
Osteoporosis, n (%)	17	(0.1)	56	(0.2)	77	(0.4)	47	(0.7)
Treatment for osteoporosis, n (%)	8	(0.1)	29	(0.1)	33	(0.2)	23	(0.4)
Selective estrogen receptor modulator	2	(0.0)	3	(0.0)	1	(0.0)	1	(0.0)
Vitamin D	7	(0.0)	25	(0.1)	31	(0.2)	23	(0.4)
Bisphosphonate	7	(0.0)	25	(0.1)	31	(0.2)	23	(0.4)
Vitamin K	0	(0.0)	0	(0.0)	1	(0.0)	0	(0.0)
Calcium	3	(0.0)	4	(0.0)	5	(0.0)	1	(0.0)

During a median follow-up of 2,500 days (interquartile range (IQR): 987–3,970), 2,753 (4.2%) experienced fractures, and the mean delta T-score was -0.03 (SD: 18.3). Among them, 36,465 (55.3%) had the same, 16,032 (24.3%) had increased, 12,556 (19.1%) had decreased, and 845 (1.3%) had a fluctuating degree of religiosity through study period. In our multivariable longitudinal analyses, the slightly religious group had significantly lower odds ratios (OR) for a fracture than did the nonreligious group in all models (OR: 0.81, 95% confidence interval (CI): 0.71 to 0.92 in model 4) (Table 2). The somewhat religious group had lower but not statistically significant ORs (OR: 0.88, 95% CI: 0.77 to 1.01 in model 4), and the very religious group had a similar incidence of fracture (OR: 1.07, 95% CI: 0.91 to 1.25 in model 4) compared to the nonreligious group. In terms of delta T score, the religious group tended to have a higher β coefficient, but the difference was not statistically significant (β coefficient: 0.10, 95% CI: -0.08 to 0.27 in model 4). There was no difference in the incidence of fracture between those who change their degree of religiosity over time and those who had same degree of religiosity over time, apart from the baseline degree of religiosity. (OR: 1.00, 95%CI: 0.92 to 1.10 for participants with increased; OR 1.00, 95%CI 0.90 to 1.10 for those with decreased; OR 1.20, 95%CI: 0.87 to 1.64 for those with fluctuated compared to those with same degree of religiosity). Table S1 shows the changes of health habits over time by the change of degree of religiosity over time. Those who had the same degree of religiosity over time (“no change” group in the change of degree of religiosity over time) tended to have same health habits. In contrast, there was no obvious trend in the change of health habits among those with increased, decreased, or both degree of religiosity overtime.

**Table 2:** Adjusted odds ratio for development of any fracture and adjusted β coefficient for change of T score from baseline by religiosity from longitudinal analyses

	Adjusted odds ratio (95% confidence interval)			
	Religiosity			
	Not religious at all	Slightly religious	Somewhat religious	Very religious
Any fracture				
Model 1	reference	**0.81 (0.72 to 0.90)**	0.91 (0.81 to 1.02)	1.07 (0.93 to 1.23)
Model 2	reference	**0.80 (0.70 to 0.91)**	0.88 (0.77 to 1.00)	1.06 (0.90 to 1.24)
Model 3	reference	**0.80 (0.70 to 0.91)**	0.88 (0.77 to 1.00)	1.06 (0.91 to 1.25)
Model 4	reference	**0.81 (0.71 to 0.92)**	0.88 (0.77 to 1.01)	1.07 (0.91 to 1.25)
Delta T score	Adjustedβ coefficient (95% confidence interval)			
Model 1	reference	-0.02 (-0.14 to 0.11)	0.01 (-0.13 to 0.14)	0.09 (-0.09 to 0.26)
Model 2	reference	-0.01 (-0.13 to 0.11)	0.02 (-0.12 to 0.15)	0.10 (-0.08 to 0.28)
Model 3	reference	-0.01 (-0.13 to 0.11)	0.02 (-0.12 to 0.15)	0.10 (-0.08 to 0.27)
Model 4	reference	-0.01 (-0.13 to 0.11)	0.02 (-0.12 to 0.15)	0.10 (-0.08 to 0.27)

Model 1 was adjusted for time variable, participants’ age and gender, and baseline T score; model 2 was adjusted for health habits (alcohol consumption, cigarette smoking and exercise) and body mass index in addition to covariates in model 1; model 3 was adjusted for comorbidities (current history of hypertension, diabetes and dyslipidemia) in addition to covariates in model 2; model 4 was adjusted for depression and its treatment status, and treatment status for osteoporosis in addition to covariates in model 3

Number in bold represents that the p value was less than 0.05

In the subanalyses by age and sex, all groups had significantly lower ORs for a fracture compared to the nonreligious reference group among older women (Table 3). The association between the degree of religiosity and a fracture among older women had a backward J shape (the very religious group had higher ORs than the slightly religious or somewhat religious group). Other subgroups, including younger women, both younger and older men, and the very religious group had higher ORs for a fracture, but most differences were not statistically significant. The results of subanalysis focusing on hip fracture and vertebral fracture as outcomes are shown in Table S2.

**Table 3:** Adjusted odds ratio for development of any fracture and adjusted β coefficient for change of T scores from baseline by religiosity stratified by age and sex from longitudinal analyses

	Adjusted odds ratio (95% confidence interval)							
	Male				Female			
	Not religious at all	Slightly religious	Somewhat religious	Very religious	Not religious at all	Slightly religious	Somewhat religious	Very religious
Age < 50 years								
Any fracture								
Model 1	Reference	0.92 (0.74 to 1.14)	1.13 (0.89 to 1.44)	1.39 (0.99 to 1.95)	Reference	0.94 (0.74 to 1.19)	1.25 (0.98 to 1.59)	**1.39 (1.02 to 1.89)**
Model 2	Reference	0.89 (0.70 to 1.13)	0.99 (0.75 to 1.30)	1.32 (0.91 to 1.90)	Reference	0.92 (0.70 to 1.22)	1.13 (0.85 to 1.49)	1.39 (0.99 to 1.96)
Model 3	Reference	0.89 (0.70 to 1.13)	0.98 (0.75 to 1.30)	1.32 (0.91 to 1.91)	Reference	0.92 (0.70 to 1.21)	1.13 (0.85 to 1.49)	1.39 (0.99 to 1.96)
Model 4	Reference	0.89 (0.70 to 1.13)	0.98 (0.75 to 1.30)	1.32 (0.91 to 1.91)	Reference	0.92 (0.70 to 1.22)	1.13 (0.86 to 1.49)	1.41 (0.99 to 1.98)
Delta T score	Adjusted β coefficient (95% confidence interval)							
Model 1	Reference	-0.01 (-0.21 to 0.20)	0.07 (-0.18 to 0.32)	0.17 (-0.20 to 0.55)	Reference	-0.03 (-0.23 to 0.18)	0.10 (-0.12 to 0.33)	0.15 (-0.17 to 0.47)
Model 2	Reference	-0.01 (-0.20 to 0.21)	0.10 (-0.15 to 0.35)	0.21 (-0.17 to 0.59)	Reference	-0.02 (-0.23 to 0.18)	0.11 (-0.12 to 0.34)	0.17 (-0.16 to 0.49)
Model 3	Reference	0.00 (-0.20 to 0.21)	0.10 (-0.16 to 0.34)	0.21 (-0.17 to 0.58)	Reference	-0.02 (-0.23 to 0.18)	0.11 (-0.12 to 0.34)	0.17 (-0.15 to 0.49)
Model 4	Reference	0.00 (-0.20 to 0.21)	0.09 (-0.16 to 0.34)	0.21 (-0.17 to 0.58)	Reference	-0.02 (-0.23 to 0.18)	0.11 (-0.12 to 0.34)	0.17 (-0.15 to 0.49)
Age ≥ 50 years								
Any fracture	Adjusted odds ratio (95% confidence interval)							
Model 1	Reference	0.89 (0.68 to 1.17)	1.00 (0.76 to 1.30)	1.13 (0.83 to 1.54)	Reference	**0.71 (0.57 to 0.88)**	**0.68 (0.56 to 0.84)**	**0.77 (0.61 to 0.97)**
Model 2	Reference	0.90 (0.66 to 1.23)	1.00 (0.73 to 1.35)	1.10 (0.78 to 1.57)	Reference	**0.63 (0.49 to 0.81)**	**0.63 (0.50 to 0.80)**	**0.74 (0.57 to 0.97)**
Model 3	Reference	0.91 (0.67 to 1.23)	1.00 (0.74 to 1.36)	1.11 (0.78 to 1.58)	Reference	**0.63 (0.49 to 0.81)**	**0.63 (0.50 to 0.80)**	**0.75 (0.57 to 0.98)**
Model 4	Reference	0.90 (0.66 to 1.23)	1.00 (0.73 to 1.35)	1.11 (0.78 to 1.58)	Reference	**0.63 (0.49 to 0.82)**	**0.63 (0.50 to 0.80)**	**0.76 (0.58 to 0.99)**
Delta T score	Adjusted β coefficient (95% confidence interval)							
Model 1	Reference	0.03 (-0.26 to 0.33)	-0.09 (-0.40 to 0.22)	0.14 (-0.24 to 0.52)	Reference	-0.20 (-0.55 to 0.15)	-0.23 (-0.58 to 0.11)	-0.24 (-0.63 to 0.15)
Model 2	Reference	0.05 (-0.25 to 0.34)	-0.06 (-0.37 to 0.25)	0.17 (-0.21 to 0.55)	Reference	-0.22 (-0.57 to 0.14)	-0.26 (-0.60 to 0.09)	-0.26 (-0.66 to 0.13)
Model 3	Reference	0.05 (-0.25 to 0.34)	-0.06 (-0.37 to 0.25)	0.17 (-0.21 to 0.55)	Reference	-0.22 (-0.57 to 0.14)	-0.26 (-0.60 to 0.09)	-0.26 (-0.66 to 0.13)
Model 4	Reference	0.04 (-0.25 to 0.34)	-0.06 (-0.37 to 0.24)	0.17 (-0.21 to 0.55)	Reference	-0.22 (-0.57 to 0.14)	-0.26 (-0.60 to 0.09)	-0.26 (-0.66 to 0.13)

Model 1 was adjusted for time variable, age and sex, and baseline T score; model 2 was adjusted for health habits (alcohol consumption, cigarette smoking and exercise) and body mass index in addition to covariates in model 1; model 3 was adjusted for comorbidities (current history of hypertension, diabetes and dyslipidemia) in addition to covariates in model 2; model 4 was adjusted for depression and its treatment status, and treatment status for osteoporosis in addition to covariates in model 3

Number in bold represents that the p value was less than 0.05

## Discussion

We demonstrated that being slightly religious is associated with a lower risk of fracture but not with changes in T-scores when compared to nonreligious individuals in all populations studied. Interestingly, somewhat or very religious people had a similar risk of fracture and delta BMD as nonreligious people in all populations studied. In addition, higher degrees of religiosity were associated with a lower risk of fracture compared to no religiosity among older women, although their T-scores were similar regardless of the degree of religiosity.

To the best of our knowledge, this study is the first to evaluate the association between the degree of religiosity and fractures and changes in BMD among a healthy population. Some previous studies may be slightly related, but most were different. Streeten et al. showed a reduced incidence of hip fracture in Old Order Amish compared to non-Amish whites in the U.S. [17] Although Old Order Amish is a religious population, the results may come from differences in lifestyle, such as very low levels of smoking or alcohol consumption, rather than the degree of religiosity. Another study from India showed different prevalences of osteopenia/osteoporosis across religions [18]. Although one study showed that regular Muslims worshipers had no difference in osteoporosis compared to nonregular worshipers, this study examined the effect of “praying” as a physical exercise rather than its religious aspects [19].

One of the potential reasons the incidence of fracture is decreased but BMD does not change in a specific religious group may be social behavioral reasons. Religious people may be more careful about accidents compared to nonreligious people in their occupation, habits, or life environment. In fact, religious people may avoid risky behaviors based on one of the common Japanese religious views—if you do a bad thing, it will come back to you- [20]. In contrast, religious people may be more active than slightly religious people [21, 22], resulting in an increased accident rate, leading to more fractures, although the increased level of exercise may have benefits for other health conditions, such as diabetes. In these ways, Japanese religious views may have both positive and negative impacts on fracture related social activities. Although we could not assume the magnitudes of each impact on the fracture, the difference of degree of religiosity may result in the different direction to fracture from the sum of each different impact. Our finding that there was no difference in delta BMD regardless of religiosity may support this hypothesis.

We also found that there was no difference in the incidence of any fracture between those who change their degree of religiosity over time and those who had same degree of religiosity over time, apart from baseline degree of religiosity. This may be simply due random error of the participants’ response over time. In fact, a similar proportion of “not religious at all” participants sometimes marked “slightly religious” over time and “very religious” participants sometimes marked “somewhat religious”. A similar phenomenon was observed for “slightly religious” and “somewhat religious” participants. Identically, the change of health habits over time had no obvious trend by the change of degree of religiosity over time.

Interestingly, the decrease in BMD was not different across religious groups. Religious people may consume more calcium, vitamin D or vitamin K. Generally, the source of animal protein in Japan mainly depended on fish hundreds of years ago for religious reasons, such as Shinto and Buddhism [23, 24]. Although current survey data about the association between religiosity and fish consumption in Japan is lacking, this tradition may have somewhat carried over, even in present days. High levels of fish consumption has been said to be associated with higher levels of vitamin D and a greater bone mass [25]. However, the effects of increased mineral intake through the diet may be insufficient for increasing BMD. In fact, calcium or vitamin D supplementation is recommended for the osteoporotic population, but there is no evidence that it increases BMD [26]. In addition, compared to cardiovascular risk factors, such as diabetes, prevention of osteoporosis may be less effective, resulting in similar BMDs across religiosity, even though religious people have a higher health awareness.

Differences in sex in the effect of religiosity on health outcomes have been reported in previous studies in other countries than Japan [27–29]. Interestingly, these studies evaluated gender differences in religiosity on health/well-being [27, 28] and cognitive function [29] and reported that the impact of religiosity on men was stronger than that on women in the U.S. and China, contrasting with our findings that religious elderly women had a lower risk of fracture in Japan. Previous authors hypothesized that this may come from increased opportunity costs of religious attendance among women in the U.S., which meant that women may have difficulty in participating in regular religious attendance due to lack of support or substitution by other social networks [27]. In contrast, because Shinto and Buddhism in Japan, which do not emphasize regular religious attendance, allow Japanese women to enjoy their religiousness, resulting in more benefit from religiosity in terms of fractures.

Our study has some limitations. First, our data contain only self-reported religiosity but not the type of religion. In addition, there was no detailed explanation for the participants about the distinction between the choices in degree of religiosity, therefore, their reported degree of religiosity totally was subjective. We cannot compare the associations across other religions. Second, we were unable to obtain complete information about fractures because a few participants were lost to follow-up. However, because being lost to follow-up may be independent of religiosity, the effect on the results would be negligible. Third, it would be reasonable to divide women into pre- or post-menopause, however, we did not have data about menopause status, so that we divided them at the age of 50, which is close to the age of menopause in Japanese women [30]. Based on these limitations, including the questions about religion, the particular nature of religion in Japan, and the lack of previous studies in this research area, additional research will be required to examine the association between the degree of religiosity and fractures.

## Conclusion

We demonstrated that slightly religious people, but not somewhat or very religious people, had a lower incidence of fracture than nonreligious people, although the T-scores were similar regardless of the degree of religiosity. We hypothesize that this population may be more careful about avoiding behaviors that might cause fractures.

## Supplementary Information

**Additional file 1.** 

**Additional file 2.** 

## Data Availability

The data underlying this article cannot be shared publicly due to patient privacy. The data will be shared on reasonable request to the corresponding author.

## Abbreviations

IQR: Interquartile range
AOR: Adjusted odds ratio
CI: Confidence interval
ICD-10: International classification of disease 10
BMD: Bone mineral density
WHO: World Health Organization
YAM: Young adult mean
SD: Standard deviation
BMI: Body mass index
GEE: Generalized estimating equation
OR): Odds ratios
