# Supplementary material for: Slight religiosity associated with a lower incidence of any fracture among healthy people in a multireligious country
Source: Biopsychosoc Med. 2023 Feb 9;17:3. doi: 10.1186/s13030-023-00265-6 (PMC9912639; doi:10.1186/s13030-023-00265-6)
Supplement: Supplementary file 1 — Additional file 1. [file 13030_2023_265_MOESM1_ESM.docx]

Table S1. The changes of health habits overtime by the change of degree of religiosity overtime

|  | Change of degree of religiosity overtime | | | |
| --- | --- | --- | --- | --- |
|  | No change | Increased | Decreased | Both increased and decreased |
| Alcohol consumption, n (%) |  |  |  |  |
| No change | 25,589 (63.5) | 8,239 (59.6) | 6,664 (59.6) | 314 (53.3) |
| Increased | 6,575 (16.3) | 2,339 (16.9) | 1,926 (17.2) | 99 (16.8) |
| Decreased | 6,963 (17.3) | 2,688 (19.5) | 2,173 (19.4) | 141 (23.9) |
| Both increased and decreased | 1,184 (2.9) | 554 (4.0) | 415 (3.7) | 35 (5.9) |
| Exercise, n (%) |  |  |  |  |
| No change | 12,521 (31.1) | 3,161 (22.9) | 2,684 (24.0) | 86 (14.6) |
| Increased | 15,402 (38.2) | 6,016 (43.5) | 4,625 (41.4) | 255 (43.3) |
| Decreased | 9,127 (22.6) | 3,119 (22.6) | 2,619 (23.4) | 158 (26.8) |
| Both increased and decreased | 3,261 (8.1) | 1,524 (11.0) | 1,250 (11.2) | 90 (15.3) |
| Smoking, n (%) |  |  |  |  |
| Never smoking overtime | 24,063 (59.7) | 7,783 (56.3) | 6,228 (55.7) | 332 (56.4) |
| Never smoked at baseline, but start smoking overtime | 773 (1.9) | 333 (2.4) | 260 (2.3) | 18 (3.1) |
| Former smoking overtime | 8,174 (20.3) | 3,025 (21.9) | 2,434 (21.8) | 134 (22.8) |
| Former smoked at baseline, but restart smoking overtime | 484 (1.2) | 192 (1.4) | 159 (1.4) | 10 (1.7) |
| Current smoking overtime | 4,255 (10.6) | 1,363 (9.9) | 1,219 (10.9) | 40 (6.8) |
| Current smoked at baseline, but stop smoking overtime | 2,562 (6.4) | 1,124 (8.1) | 878 (7.9) | 55 (9.3) |
