# Supplementary material for: Slight religiosity associated with a lower incidence of any fracture among healthy people in a multireligious country
Source: Biopsychosoc Med. 2023 Feb 9;17:3. doi: 10.1186/s13030-023-00265-6 (PMC9912639; doi:10.1186/s13030-023-00265-6)
Supplement: Supplementary file 2 — Additional file 2. [file 13030_2023_265_MOESM2_ESM.docx]

Table S2. Adjusted odds ratio for development of hip and vertebral fractures by religiosity stratified by gender among elderly (age≥50 years) from longitudinal analyses.

|  | Adjusted odds ratio  (95% confidence interval) | | | | | | | |
| --- | --- | --- | --- | --- | --- | --- | --- | --- |
|  | Male | | | | Female | | | |
|  | Not religious at all | Slightly religious | Somewhat religious | Religious | Not religious at all | Slightly religious | Somewhat religious | Religious |
| Hip or vertebral fractures | n=100 | | | | n=177 | | | |
| Model 1 | Reference | 0.48 (0.22 to 1.03) | 1.02 (0.53 to 1.98) | 1.10 (0.53 to 2.28) | Reference | 0.76 (0.43 to 1.35) | 0.84 (0.50 to 1.41) | 0.85 (0.49 to 1.47) |
| Model 2 | Reference | 0.84 (0.21 to 3.36) | 1.82 (0.52 to 6.37) | 2.27 (0.61 to 8.46) | Reference | 0.68 (0.24 to 1.91) | 1.00 (0.41 to 2.48) | 1.49 (0.59 to 3.75) |
| Model 3 | Reference | 0.84 (0.21 to 3.37) | 1.83 (0.52 to 6.43) | 2.28 (0.61 to 8.54) | Reference | 0.70 (0.25 to 1.99) | 1.06 (0.43 to 2.62) | 1.58 (0.63 to 4.00) |
| Model 4 | Reference | 0.82 (0.20 to 3.29) | 1.78 (0.51 to 6.28) | 2.15 (0.57 to 8.10) | Reference | 0.72 (0.26 to 2.06) | 1.07 (0.43 to 2.65) | 1.63 (0.65 to 4.12) |
| Vertebral fracture | n=83 | | | | n=142 | | | |
| Model 1 | Reference | 0.50 (0.22 to 1.14) | 1.13 (0.56 to 2.31) | 0.80 (0.34 to 1.89) | Reference | 0.93 (0.49 to 1.79) | 0.97 (0.54 to 1.76) | 0.92 (0.49 to 1.74) |
| Model 2 | Reference | 0.72 (0.17 to 3.03) | 1.80 (0.51 to 6.36) | 1.23 (0.29 to 5.31) | Reference | 0.81 (0.27 to 2.43) | 1.16 (0.44 to 3.07) | 1.62 (0.59 to 4.39) |
| Model 3 | Reference | 0.72 (0.17 to 3.04) | 1.81 (0.51 to 6.39) | 1.23 (0.29 to 5.32) | Reference | 0.85 (0.28 to 2.54) | 1.22 (0.46 to 3.24) | 1.72 (0.63 to 4.69) |
| Model 4 | Reference | 0.70 (0.17 to 2.95) | 1.76 (0.50 to 6.25) | 1.16 (0.27 to 5.02) | Reference | 0.88 (0.29 to 2.64) | 1.24 (0.46 to 3.30) | 1.78 (0.65 to 4.86) |
| Hip fracture | n=17 | | | | n=35 | | | |
| Model 1 | Reference | 0.39 (0.05 to 2.75) | 0.49 (0.08 to 2.97) | 2.43 (0.50 to 11.9) | Reference | 0.33 (0.09 to 1.22) | 0.51 (0.18 to 1.47) | 0.67 (0.21 to 2.07) |
| Model 2 | Reference | 0.15 (0.02 to 1.39) | **0.11 (0.01 to 0.96)** | - | Reference | - | 0.22 (0.01 to 3.71) | 0.61 (0.05 to 7.38) |
| Model 3 | Reference | 0.15 (0.02 to 1.37) | **0.11 (0.01 to 0.98)** | - | Reference | - | 0.24 (0.01 to 4.28) | 0.74 (0.06 to 9.00) |
| Model 4 | Reference | 0.16 (0.17 to 1.50) | 0.12 (0.01 to 1.11) | - | Reference | - | 0.24 (0.01 to 4.30) | 0.72 (0.06 to 8.76) |

Model 1 was adjusted for time variable, participants’ age and gender, and baseline T score; model 2 was adjusted for health habits (alcohol consumption, cigarette smoking and exercise) and body mass index in addition to covariates in model 1; model 3 was adjusted for comorbidities (current history of hypertension, diabetes and dyslipidemia) in addition to covariates in model 2; model 4 was adjusted for depression and its treatment status, and treatment status for osteoporosis in addition to covariates in model 3.

Number in bold represents that the p value was less than 0.05.
